# The *β*-Lactamase Gene Profile and a Plasmid-Carrying Multiple Heavy Metal Resistance Genes of *Enterobacter cloacae*

**DOI:** 10.1155/2018/4989602

**Published:** 2018-12-20

**Authors:** Chongyang Wu, Chaoqin Lin, Xinyi Zhu, Hongmao Liu, Wangxiao Zhou, Junwan Lu, Licheng Zhu, Qiyu Bao, Cong Cheng, Yunliang Hu

**Affiliations:** ^1^The Second Affiliated Hospital and Yuying Children's Hospital of Wenzhou Medical University, Wenzhou, Zhejiang 325000, China; ^2^School of Laboratory Medicine and Life Sciences/Institute of Biomedical Informatics, Wenzhou Medical University, Wenzhou 325035, China; ^3^College of Medicine and Health, Lishui University, Lishui 323000, China

## Abstract

In this work, by high-throughput sequencing, antibiotic resistance genes, including class A (*bla*_CTX-M_, *bla*_Z_, *bla*_TEM_, *bla*_VEB_, *bla*_KLUC_, and *bla*_SFO_), class C (*bla*_SHV_, *bla*_DHA_, *bla*_MIR_, *bla*_AZECL-29_, and *bla*_ACT_), and class D (*bla*_OXA_) *β*-lactamase genes, were identified among the pooled genomic DNA from 212 clinical *Enterobacter cloacae* isolates. Six *bla*_MIR_-positive *E. cloacae* strains were identified, and pulsed-field gel electrophoresis (PFGE) showed that these strains were not clonally related. The complete genome of the *bla*_MIR_ *-*positive strain (Y546) consisted of both a chromosome (4.78 Mb) and a large plasmid pY546 (208.74 kb). The extended-spectrum *β*-lactamases (ESBLs) (*bla*_SHV-12_ and *bla*_CTX-M-9a_) and AmpC (*bla*_MIR_) were encoded on the chromosome, and the pY546 plasmid contained several clusters of genes conferring resistance to metals, such as copper (*pco*), arsenic (*ars*), tellurite (*ter*), and tetrathionate (*ttr*), and genes encoding many divalent cation transporter proteins. The comparative genomic analyses of the whole plasmid sequence and of the heavy metal resistance gene-encoding regions revealed that the plasmid sequences of *Klebsiella pneumoniae* (such as pKPN-332, pKPN-3967, and pKPN-262) shared the highest similarity with those of pY546. It may be concluded that a variety of *β*-lactamase genes present in *E. cloacae* which confer resistance to *β*-lactam antibiotics and the emergence of plasmids carrying heavy metal resistance genes in clinical isolates are alarming and need further surveillance.

## 1. Introduction

Bacteria of the *Enterobacter cloacae complex* (*ECC*), which comprises six species, namely, *E. cloacae*, *E. asburiae*, *E. hormaechei*, *E. kobei*, *E. ludwigii*, and *E. nimipressuralis* [1], are widely distributed in nature. As pathogens, *ECC* species are highly adapted to the environment and are able to contaminate hospital medical devices. Currently, *E. cloacae* and *E. hormaechei* are most frequently isolated from human clinical specimens, and *E. cloacae* is among the *Enterobacter* sp. that have most commonly caused nosocomial infections in the last decade [2]. Furthermore, *E. cloacae* has assumed clinical importance and has emerged as a major human pathogen; it accounts for up to 5% of hospital-acquired bacteremia cases, 5% of nosocomial pneumonia cases, 4% of nosocomial urinary tract infections, and 10% of postsurgical peritonitis cases [3].

Owing to the low-level but inducible expression of a chromosomal *ampC* gene encoding the AmpC *β*-lactamase, *E. cloacae* is intrinsically resistant to ampicillin, amoxicillin-clavulanate, and first-generation cephalosporins [4]. Generally, the resistance of *E. cloacae* to third-generation cephalosporins is caused by its overproduction of the AmpC *β*-lactamases when the production of this cephalosporinase is inducible in the presence of strong *β*-lactam antibiotics (cefoxitin and imipenem); thus, treatment with third-generation cephalosporins may promote the development of AmpC-overproducing mutants. AmpC-producing organisms become resistant to almost all *β*-lactam antibiotics, with the exception of cefepime, cefpirome, and carbapenems. Most chromosomal *ampC* genes are inducible in the presence of certain agents such as cefoxitin and imipenem. Inducible AmpC expression is regulated by AmpR in the presence of two other gene products, namely, AmpD and AmpG. The regulation of AmpC production has been historically understood to require three proteins: AmpG, a plasma membrane-bound permease; AmpD, a cytosolic peptidoglycan-recycling amidase; and AmpR, the transcriptional regulator of AmpC. Derepression has been associated previously with structural defects within the *ampD* gene or with decreased *ampD* expression. Derepression represents the inability of AmpR to keep AmpC expression at constitutively low wild-type levels [5]. As a result, the AmpC enzyme confers resistance to third-generation cephalosporins and is not inhibited by common *β*-lactamase inhibitors. However, fourth-generation cephalosporins still retain activity against most *Enterobacteriaceae* strains.

In addition to therapeutic antibiotic agents, a large number of other chemical substances with antibacterial activities, such as heavy metals and detergents, are used in human health care and agricultural practices. Recently, concerns have been raised regarding coselection for antibiotic resistance among bacteria exposed to disinfectants and heavy metals (particularly copper, zinc, and mercury) used in some livestock species as growth promoters and therapeutic agents [6]. *Enterobacteriaceae* (including *E. coli*, *K. pneumoniae*, and *E. cloacae*) are highly adept at acquiring resistance genes to all disinfectants, heavy metals, and antibiotics through horizontal gene transfer between different bacteria within the environment; such genes include extended-spectrum beta-lactamases (ESBLs), copper and arsenic resistance systems (the *pco and ars* operons), and enzymes that hydrolyze cephalosporins (AmpC enzymes) [7, 8]. Many gram-negative organisms (such as *E. coli*, *E. cloacae*, and *K. pneumoniae*) encode broad-substrate efflux pumps [6, 7, 9], and a variety of multidrug pumps that have activity against disinfectants are similarly encoded by some gram-positive organisms, including *Staphylococcus aureus* [9, 10]. Besides the efflux pumps, other mechanisms such as chemisorption facilitating cadmium to bind to the bacterial surface also played a role in the heavy metal (cadmium) resistance [11]. Alternatively, in both gram-positive and gram-negative bacteria, mechanisms of acquired resistance to disinfectants may be associated with efflux pump-encoding genes introduced on mobile genetic elements or, in gram-negative bacteria, with mutations causing the constitutive overexpression of efflux pumps. Compared with antibiotics and disinfectants, heavy metals (copper, arsenic, zinc, and mercury) are very persistent in the environment and may accumulate in soil, water, and sediments from agricultural practices as well as from other sources such as aquacultural and industrial effluents [12]. Like the mechanisms of resistance to disinfectants, efflux pumps can expel heavy metal ions; such pumps include elements of the *czc* system, which encodes a pump for zinc, cobalt, and cadmium, and *pcoA*, which is an element of a copper extrusion system (W. [13]).

In this study, we identified *β*-lactamase genes in 212 clinical *E. cloacae* isolates and sequenced a *bla*_MIR_ gene-carrying strain. Molecular analyses were performed to analyze the function of the resistance genes, and a comparative genomics analysis was conducted to elucidate the potential horizontal gene transfer patterns of genes related to both antibiotic and heavy metal resistance between bacteria of different species or genera. Our analysis revealed the distinct structure of a large plasmid carrying multiple clusters of heavy metal resistance genes that, to our knowledge, have not been described previously in *E. cloacae*.

## 2. Materials and Methods

### 2.1. Bacterial Strain Collection, Genomic DNA Extraction, and High-Throughput Sequencing

A total of 212 nonduplicate clinically significant *E. cloacae* strains were isolated from the First Affiliated Hospital of Wenzhou Medical University (Zhejiang, China) between 2008 and 2012. These isolates were identified by a VITEK 60 microbial autoanalyzer (bioMérieux, Lyon, France). Bacteria and plasmids used in this study are listed in Table 1. Among the isolates, 31, 36, 43, 32, and 70 strains were isolated in the years 2008, 2009, 2010, 2011, and 2012, respectively. All strains were resistant to a minimum of one or two antibiotics. For pooled genomic DNA sequencing, each clinical strain was incubated in 5 mL of Luria-Bertani (LB) broth at 37°C for approximately 16 h to obtain a concentration equivalent to an optimum optical density (OD_600_ = 1.5 ± 0.2). The cultures were pooled, and genomic DNA was extracted from 100 mL of the mixed bacteria using an AxyPrep Bacterial Genomic DNA Miniprep Kit (Axygen Scientific, Union City, CA, USA). The pooled genomic DNA was sequenced with a HiSeq 2500 DNA sequencer at Annoroad Gene Technology Co. Ltd. (Beijing, China). Reads derived from the HiSeq 2500 sequencing were initially assembled de novo with SOAPdenovo software to obtain contigs of the genome sequences. Glimmer software (http://ccb.jhu.edu/software/glimmer) was used to predict protein-coding genes with potential open reading frames (ORF) > 150 bp in length. BLASTX (https://blast.ncbi.nlm.nih.gov) was used to annotate the predicted protein-coding genes against the nonredundant protein database with an e-value threshold of 1*E*-5.

### 2.2. Collection of Reference Sequences for Resistance Genes and Mapping of Sequencing Reads to the Reference Genes

The nucleotide sequences of all *β*-lactamase genes, including those encoding for Ambler class A, B, C, and D *β*-lactamases, were obtained from the GenBank nucleotide database, and the high-throughput sequencing reads were mapped to the reference sequences of the *β*-lactamase genes as previously described [14]. The relative abundance (sequencing depth) of a certain gene was calculated as the cumulative nucleotide length of the mapped short reads on the gene divided by the gene size.

### 2.3. Screening of *β*-Lactamase Gene-Positive Strains and Cloning and Phylogenetic Analysis of *bla*_MIR_ Genes

The *E. cloacae* strains were screened by PCR amplification for the presence of *β*-lactamase genes as previously described [15–18]. The primers used for the cloning of complete ORFs contained a pair of flanking restriction endonuclease adapters (*BamH*I for the forward primers and *Hind*III for the reverse primers) and were designed using the Primer Premier 5.0 software package; the primers are shown in Table 2. Genomic DNA was extracted from each of the 212 clinical *E. cloacae* isolates using the AxyPrep Bacterial Genomic DNA Miniprep kit (Axygen Scientific, Union City, CA, USA) and was used as the template for PCR amplification. Positive amplification products were verified by sequencing with an ABI 3730 automated sequencer (Shanghai Sunny Biotechnology Co. Ltd., Shanghai, China), and the sequencing results were compared with Basic Local Alignment Search Tool (BLAST) algorithms (https://blast.ncbi.nlm.nih.gov/blast.cgi). The amplicons of the *bla* ORFs were digested with the corresponding restriction endonucleases and ligated into the pET-28a vector with a T4 DNA ligase cloning kit (Takara Bio Inc., Dalian, China). The recombinant plasmid was transformed into competent *E. coli* BL21 cells using the calcium chloride method and cultured on LB agar plates supplemented with ampicillin (200 *μ*g/mL), and the cloned ORFs were confirmed by sequencing. For the phylogenetic analysis, all *bla*_MIR_ gene sequences were collected from the NCBI nucleotide database using *bla*_MIR_ as the key search term. A phylogenetic tree of the MIR amino acid sequences from both the database and this work was reconstructed by the maximum likelihood method, and the resulting trees were analyzed with bootstrap values of 100 replicates using MEGA 6.0 (https://www.megasoftware.net/).

### 2.4. Antimicrobial Susceptibility Testing

Antimicrobial susceptibility testing was conducted for all tested antibiotics by the agar dilution method, and the minimum inhibitory concentrations (MICs) were interpreted based on the Clinical and Laboratory Standards Institute (CLSI) breakpoint criteria (CLSI, 2018) (https://clsi.org/standards/products/packages/m02-m07-m100-package/). Strain ATCC 25922 was used as the quality control strain. The 14 antibiotics (or antibiotics combination) used in this work were cephamycins (cefoxitin and cefminox), semisynthetic broad-spectrum penicillins (ampicillin and piperacillin), a first-generation cephalosporin (cefazolin), third-generation cephalosporins (ceftazidime, cefoperazone, cefotaxime, and ceftriaxone), a fourth-generation cephalosporin (cefoselis), a monobactam (aztreonam), an aminoglycoside (kanamycin), and combinations of antibiotics with *β*-lactamase inhibitors (piperacillin/tazobactam and imipenem/cilastatin sodium hydrate) (Table 3).

### 2.5. Whole Genome Sequencing (WGS) of Y546 and Comparative Genomics Analysis

The *E. cloacae* Y546 genomic DNA was extracted with the AxyPrep Bacterial Genomic DNA Miniprep Kit (Axygen Scientific, Union City, CA, USA) and sequenced with Illumina HiSeq 2500 and Pacific Biosciences sequencers at Annoroad Gene Technology Co. Ltd. (Beijing, China). Reads derived from HiSeq 2500 sequencing were initially assembled de novo with SOAPdenovo software to obtain genome sequence contigs. Reads of approximately 10-20 kb in length from the Pacific Biosciences sequencing were mapped onto the primary assembly for contig scaffolding. Gaps were filled either by remapping the short reads from the HiSeq 2500 sequencing or by sequencing the gap PCR product. Potential ORFs were predicted and annotated using Glimmer3 (http://www.cbcb.umd.edu/software/glimmer) and BASys [19], respectively. GC view software was used to construct the basic genomic features. Annotations were revised using UniProt (http://www.uniprot.org/) and BLAST (https://blast.ncbi.nlm.nih.gov/blast.cgi). Plasmid replicons and plasmid incompatibility groups were predicted using Plasmid Finder (https://cge.cbs.dtu.dk//services/PlasmidFinder/). Furthermore, the multilocus sequence typing (MLST) database for *E. cloacae* (https://pubmLst.org/ecloacae/) was used to determine the sequence type of *E. cloacae* Y546.

The plasmid and chromosomal genomic sequences used in this study for the whole genome comparative analysis were downloaded from the NCBI database (http://www.ncbi.nlm.nih.gov). The plasmid and chromosome sequences were selected based on the comparison of the whole genome sequence (pY546) against the sequences of plasmids and chromosomes available in the NCBI database; a cutoff value (max score) of approximately 8700 was defined. For the comparative analysis of the heavy metal gene cluster regions on the pY546 plasmid, the sequences containing corresponding gene clusters with sequence identities of ≥80% with respect to those encoded on pY546 were obtained from the NCBI nucleotide database by BLASTn algorithms. Multiple sequence alignments were performed using MAFFT [20]. Comparisons of the nucleotide sequences were made using BLASTn. Insertion sequences were predicted using ISfinder [21]. Orthologous groups of genes from plasmids or chromosomes were identified using BLASTp and Inparanoid [22]. Additional bioinformatics software was written using Python (https://www.python.org/) and Biopython [23].

### 2.6. Pulsed-Field Gel Electrophoresis (PFGE)

The clonal relatedness of MIR-producing *E. cloacae* isolates was assessed by *Xba*I (Takara Bio Inc., China) PFGE. Briefly, genomic DNA fragments were resolved on a 1% agarose (SeaKem Gold Agarose, Lonza) gel at 14°C, and electrophoresis was conducted at 6 V/cm using a CHEF PFGE instrument (Bio-Rad, USA) at a pulse time gradient of 2.25-55.5 s and a total run time of 18 h. *Salmonella enterica* serovar H9812 was used as a control. Cluster analysis was performed using an unweighted pair-group method with arithmetic (UPGMA) means. Isolates were allocated into genetic similarity clusters using a similarity cutoff value of 80% [24].

### 2.7. Nucleotide Sequence Accession Numbers

The sequences of the chromosome and the plasmid of Y546 and the *bla*_MIR_ genes in this work have been submitted to NCBI GenBank with accession numbers of CP032916 (Y546), CP032915 (pY546), MK033024 (*bla*_MIR-CG34_), MK033023 (*bla*_MIR-Y490_), MK033025 (*bla*_MIR-CG76_), MK033026 (*bla*_MIR-Y546_), MK033021 (*bla*_MIR-CG85_), and MK033022 (*bla*_MIR-Y482_), respectively.

## 3. Results

### 3.1. Mapping and Screening Results for the *β*-Lactamase Genes in the Sequenced Bacteria

A total of 75 *β*-lactamase gene sequences were collected from the database (Supplementary Table S1). The pooled genomic DNA sequences of the 212 isolated strains generated approximately 34.1 gigabases. All reads ranged from 100 to 110 nucleotides in length. The mapping of the sequencing reads onto the reference sequences yielded the identification of resistance genes; in addition, the quantity of mapped reads on a specific reference could suggest the relative abundance of the reads in the sequenced samples. This analysis showed that the samples contained a total of 12 hits related to *β*-lactamase resistance genes. The most abundant gene was *bla*_TEM_, which had a sequence depth of 466.15 (Supplementary Table S2). The other genotypes with a greater abundance were *bla*_SHV_, *bla*_DHA_, and *bla*_CTX-M_ (especially the *bla*_CTX-M-9_ and *bla*_CTX-M-1_ subtypes), while the genotypes with a lower abundance were *bla*_Z_, *bla*_VEB_, *bla*_KLUC_, *bla*_MIR_, *bla*_SFO_ *, bla*_AZECL_, *bla*_OXA_, and *bla*_ACT_. The screening results for the *bla*_VEB_, *bla*_Z_, *bla*_AZECL_, and *bla*_MIR_ genotypes revealed that among the 212 strains, only 0.47% (1/212; Y412), 0.94% (2/212; CG3 and CG4), 0.94% (2/212; Y411 and CG90), and 2.83% (6/212; CG34, Y490, CG76, Y546, CG85, and Y482) carried *bla*_VEB_, *bla*_Z_, *bla*_AZECL_, and *bla*_MIR_, respectively.

### 3.2. Cloning and Functional Detection of the Resistance Genes

Fourteen antimicrobial agents were used to detect the MIC levels of the *bla*_MIR_ *-*positive wild-type strains and the recombinant strains expressing the cloned *bla*_MIR_ genes (pET28a-*bla*_MIR_/BL21). The MICs of the 14 antimicrobial agents against these strains are shown in Table 3. The MICs for all 6 *bla*_MIR_-positive wild-type strains (CG34, Y490, CG76, Y546, CG85, and Y482) demonstrated that they were resistant to 4 commonly used broad-spectrum beta-lactam antibiotics, including ampicillin, a first-generation cephalosporin (cefazolin), and cephamycins (cefmenoxime and cefoxitin), and *E. cloacae* CG76 displayed higher resistance levels than the other strains to all antibiotics tested. Like the host wild-type strains, the 6 recombinant strains expressing the cloned *bla*_MIR_ genes (pET28a-*bla*_MIR_/BL21) were resistant to ampicillin, cefazolin, cefmenoxime, and cefoxitin. In addition, the recombinants with two other extended-spectrum *β*-lactamase (ESBL) genes, namely, *bla*_SHV-12_ and *bla*_CTX-M-9a_, encoded on the Y546 chromosome displayed higher hydrolytic activity against these four *β*-lactam antibiotics.

### 3.3. Clonal Relatedness of the *bla*_MIR_-Positive Strains and Genotypes and the Phylogenetic Tree Analysis of the *bla*_MIR_ Genes

**Figure 1:**
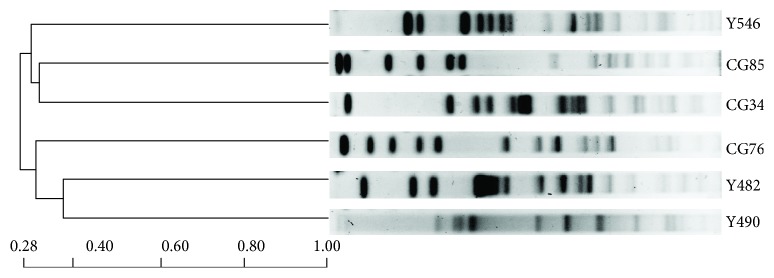
Pulsed-field gel electrophoresis (PFGE) analysis of the 6 *bla*_MIR_-positive *E. cloacae* isolates. PFGE result showed that all 6 *bla*_MIR_-positive isolates had a totally distinct PFGE pattern.

**Figure 2:**
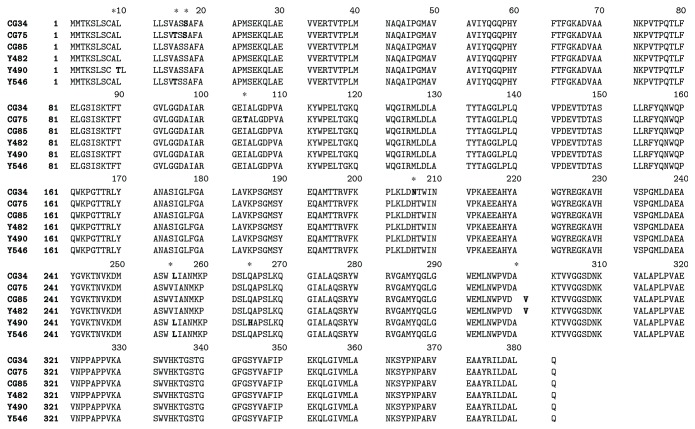
Comparison of the MIR amino acid sequences from 6 *E. cloacae* isolates.

**Figure 3:**
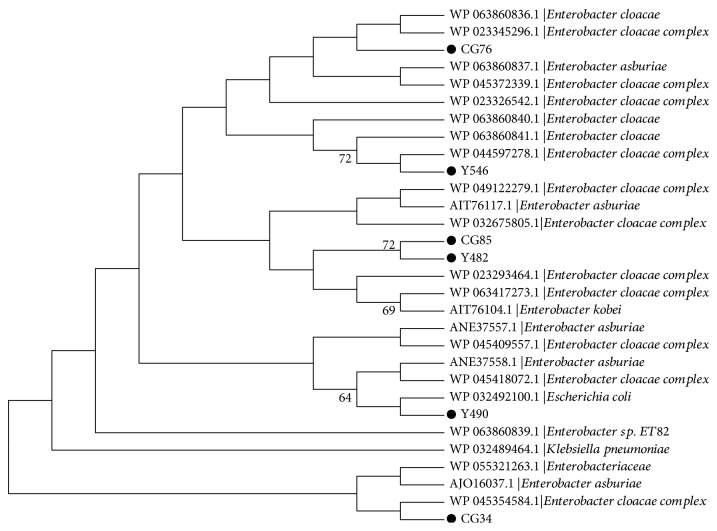
Phylogenetic tree of 30 MIR amino acid sequences. The “●” symbols indicate the MIRs of this study.

All 6 *bla*_MIR_-positive strains (Y490, Y482 CG34, CG76, CG85, and Y546) had distinct *Xba*I PFGE patterns (Figure 1), indicating that the prevalence of *bla*_MIR_-positive isolates was caused by disseminated gene transfer. Sequencing results showed that the *bla*_MIR_ ORFs from strains CG85 and Y482 belonged to the *bla*_MIR-17_ genotype, while the ORFs from strains CG34, CG76, Y490, and Y546 matched *bla*_MIR-5_, *bla*_MIR-21_, *bla*_MIR-3_, and *bla*_MIR-20_, respectively. These ORFs had 99% amino acid similarity to their respective reference sequences. To further analyze the evolutionary relationship of the 6 *bla*_MIR_ genes identified in this work with other *bla*_MIR_ genes, we performed a multiple sequence alignment on a total of 30 *bla*_MIR_ variants including the 6 *bla*_MIR_ genes identified in this study. The multiple-sequence alignment identified the Pro380Leu variant in *bla*_MIR-Y546_, *bla*_MIR-Y482_, and *bla*_MIR-Y490_ and the Ala381Gln variant in *bla*_MIR-CG76_, *bla*_MIR-CG85_, *bla*_MIR-Y546_, *bla*_MIR-Y482_, and *bla*_MIR-Y490_. The Asn206His variant was identified only in *bla*_MIR-CG34_ (Figure 2). The phylogenetic analysis (Figure 3) showed that with the exception of 2 sequences from CG85 and Y482 that had the same amino acid sequence and were located in the same branch, the 6 MIR proteins were located in unique branches.

### 3.4. General Features of the Y546 Genome

The genome of *E. cloacae* strain Y546 consists of a chromosome and a plasmid (pY546); the general features of the Y546 genome are shown in Table 4. The chromosome is 4.78 Mb in length, harbors 4312 ORFs, and has an average GC content of 56.02%. In addition to an *ampC* gene *bla*_MIR_, the chromosome also encodes two other extended-spectrum *β*-lactamase (ESBL) genes, namely, *bla*_CTX-M-9_ and *bla*_SHV-12_. MLST determined that *E. cloacae* strain Y546 contains the *leuS*-90, *rpoB*-20, *gyrB*-127, *dnaA*-120, *fusA*-25, and *rplB*-12 alleles and belongs to the sequence type ST466. The pY546 plasmid, an IncHI1B plasmid, is 208,740 bp in length and encodes 232 ORFs (Supplementary Table S3), of which 56.89% (132/232) encode proteins with known functions, and it contains a number of accessory modules identified at different sites within the backbone. pY546 contained an incomplete copper resistance operon (*pcoBCDE/cusRS*, ORF91-96) and several clusters of genes related to resistance to other metals, including arsenic (*arsABCDR*, ORF100-105), tetrathionate (*ttrABCDRS*, ORF142-146) and tellurite (*terCDEF*, ORF153-156). This plasmid also encodes numerous metallic ion metabolism and transfer proteins, such as a potassium transporter (*Kef*, ORF109), a fluoride ion transporter (*CrcB*, ORF113), divalent cation transporters (ORF111 and ORF112), and voltage-gated chloride channel proteins (ORF120 and ORF121). In addition, TonB-dependent receptors (TBDRs, ORF12-14), which are involved in the uptake of essential nutrients, are identified in pY546.

### 3.5. Comparative Genomic Analysis of the pY546 Plasmid Genome

**Figure 4:**
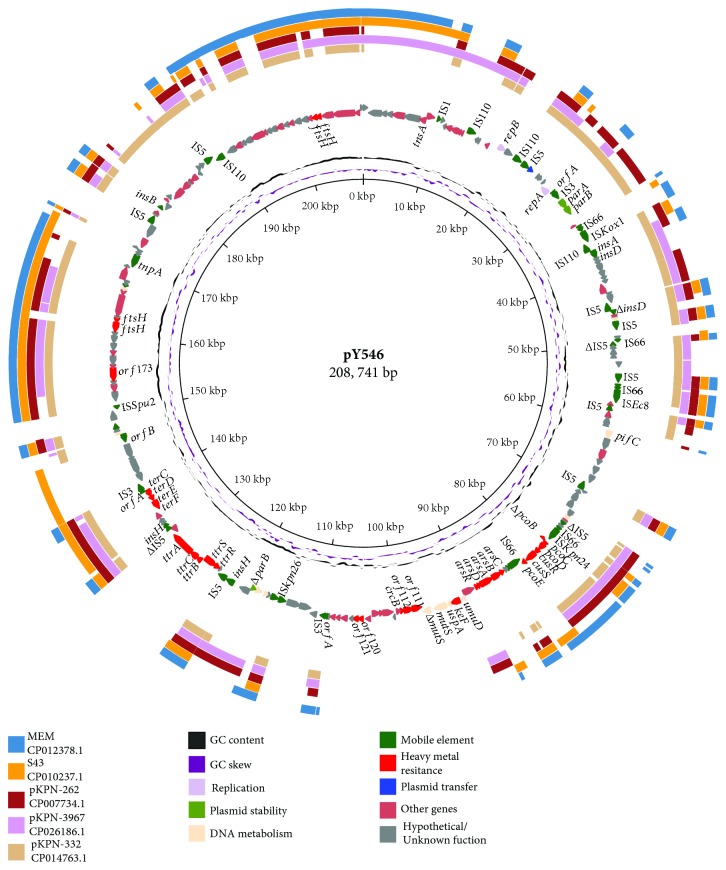
Complete sequence of the pY546 plasmid and comparative genomic analysis of the pY546 plasmid sequence with other sequences. The circles (from innermost to outermost) represent (i) the scale in kb; (ii) the cumulative GC skew; (iii) the GC content; (iv) the annotated coding sequences with selected genes indicated according to the gene function: heavy metal resistance genes in red arrows, transposase genes, IS elements in bottle-green arrows, and hypothetical proteins in dark gray arrows; (v) circles (from inside to outside) representing three homologous plasmids (pKPN-332, CP014763.1; pKPN-3967, CP026186.1; and pKPN-262, CP007734.1) and two chromosome fragments (S43, CP010237.1, and MEM, CP012378.1), respectively.

A total of six sequences having greater than 40% nucleotide sequence identity to the pY546 sequence were retrieved from GenBank. Four of these were plasmid sequences, namely, pKPN-332 of *K. pneumoniae* strain KPNIH39 (49%, CP014763.1), pKPN-3967 of *K. pneumoniae* strain KPNIH49 (47%, CP026186.1), plasmid unnamed 1 of *K. pneumoniae* strain KSB2_1B (44%, CP024507.1), and pKPN-262 of *K. pneumoniae* subsp. pneumoniae KPNIH27 (44%, CP007734.1). The other two were chromosome sequences of *E. coli* S43 (41%, CP010237.1) and *E. coli* MEM (40%, CP012378.1) (Figure 4). Comparative genomics analysis showed that pY546 is approximately 80-100 kb smaller than any of the three named plasmids (pKPN-332, pKPN-3967, and pKPN-262). The sequences of 102 genes (43.4%, 102/235) on pY546 showed high similarity (>90%) with those on each of the three named plasmids. These plasmids shared a conserved backbone sequence with pY546; the backbone included the replication initiation gene (*rep*A), stable maintenance genes (*par*AB or *sop*AB), DNA mismatch repair system genes (*mutS*), and so on. On the other hand, all these plasmids possessed their own variable regions, which mainly included heavy metal (*cop*, *ars*, and *ter*) resistance gene clusters and hypothetical genes. The tetrathionate resistance genes (*ttrABCRS*) encoded on pY546 were not present in the three named plasmids. The named plasmids also had multiple copies of common mobile elements, such as transposons and insertion elements (IS). In the two *E. coli* (S43 and MEM) chromosome sequences, the *pco* gene cluster (*pcoABCDRSE*) was detected in MEM but not in S43. Nevertheless, *arsBCD*, which is a part of the arsenic resistance determinants, was present in both S43 and MEM. The tetrathionate resistance genes were unique to pY546, while the chromosome of S43 carried the *ter* operon, but the MEM chromosome did not (Figure 4). The *ter* operon *terCDEF* was detected and in the same orientation in the three plasmids pKPN-332, pKPN-3967, and pKPN-262.

### 3.6. Comparative Analysis of Copper and Arsenic Resistance Gene Regions on the pY546 Plasmid

**Figure 5:**
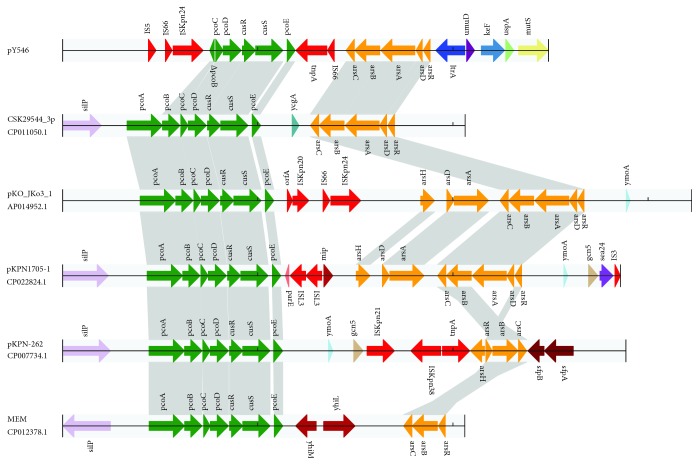
Comparative analysis of the copper and arsenic resistance gene clusters of pY546 and other sequences from different bacteria. The homologous gene clusters among plasmids pY546, CSK29544_3P (CP011050.1), pKO_JKo3_1 (AP014952.1), pKPN1705-1 (CP022824.1), pKPN-262 (CP007734.1), and MEM (CP012378.1) with the copper resistance gene clusters in bottle green, and the arsenic resistance gene cluster in orange. Annotated coding sequences are displayed as arrows. Coding sequences are colored based on their assigned gene functions.

Comparative analysis of an 8.7 kb fragment of pY546 encoding both copper (*pco*) and arsenic (*ars*) operons showed that the 5 sequence fragments with the highest similarity to that of pY546 were 4 fragments from the pKO_JKo3_1 plasmid of *Klebsiella oxytoca* JKo3 (100%, AP014952.1), the pKPN1705-1 plasmid of *Klebsiella quasivariicola* KPN1705 (100%, CP022824.1), the CSK29544_3p plasmid of *Cronobacter sakazakii* ATCC 29544 (100%, CP011050.1), and the pKPN-262 plasmid of *Klebsiella pneumoniae* subsp. *pneumoniae* KPNIH27 (82%, CP007734.1) and 1 fragment from the chromosome of *Escherichia coli* MEM (CP012378.1, 82%) (Figure 5). The fragment sharing the highest sequence identity with that of pY546 (from *E. cloacae*) was in the CSK29544_3p plasmid of *Enterobacter sakazakii.* All sequences except for pY546 contained the complete copper (*pco*) operon structure. pY546, however, contained an incomplete copper (*pco*) operon with a truncated *pcoB* (△*pcopB*) gene and without *pcoA* gene. Four (pY546, CSK29544_3p, pKO_Jko3_1, and pKPN1705-1) sequences contained the complete *ars* operon gene clusters. Moreover, the latter two plasmids (pKO_Jko3_1 and pKPN1705-1) contained an additional functional gene, namely, *arsH*, which encoded an organoarsenical oxidase (NADPH-dependent FMN reductase) [25] and conferred resistance to trivalent forms of organoarsenic compounds. Two of the plasmids (pKO_Jko3_1 and pKPN1705-1) were also identified to contain two copies of *arsA and arsD* with inverted orientations. The CSK29544_3p plasmid contained the same gene arrangement and content as pY546, but pY546 and CSK29544_3p contained fewer genes than the other two plasmids (pKO_Jko3_1 and pKPN1705-1). The pY546 and CSK29544_3p plasmids lacked *arsH* and contained only one copy each of *arsAD*, which was oriented oppositely in these two plasmids. On the other hand, the *arsBCRH* gene cluster was identified in pKPN-262, while the MEM chromosome contained *arsBCR* but lacked *arsH*.

## 4. Discussion

The production of *β*-lactamases is the predominant *β*-lactam resistance mechanism in gram-negative bacteria. The Ambler molecular classification categorizes these *β*-lactamases into four enzyme classes, namely, A, B, C, and D. Class A, C, and D enzymes all possess an active site serine, whereas class B *β*-lactamases are metalloenzymes with a Zn^2+^ ion(s) in the active site [26]. In this study, a total of 12 *β*-lactamase-encoding genes, including 7 class A *β*-lactamase genes (*bla*_SHV_, *bla*_CTX-M_, *bla*_Z_, *bla*_VEB_, *bla*_KLUC_, *bla*_SFO_, and *bla*_TEM_), 4 class C *β*-lactamase genes (*bla*_MIR_, *bla*_DHA_, *bla*_ACT_, and *bla*_AZECL-29_), and 1 class D *β*-lactamase gene (*bla*_OXA_) were identified in 212 *E. cloacae* isolates from a teaching hospital in South China. Over the past years, a variety of metalloenzymes (NDM- and IMP-type) have been found in *E. cloacae* and have contributed to infectious outbreaks in China [27] and Japan [28]. However, we did not detect any genes encoding class B metalloenzymes in these 212 *E. cloacae* isolates.

The class A enzymes are regarded as extended-spectrum *β*-lactamases (ESBLs) that can hydrolyze extended-spectrum cephalosporins; they are inhibited by clavulanic acid and are spreading widely among *Enterobacteriaceae*. The CTX-M enzymes are replacing the SHV and TEM enzymes as the most prevalent type of ESBL in *Enterobacteriaceae* [29, 30]. Additional clinically relevant types of ESBLs include the VEB, PER, GES, TLA, IBC, SFO-1, BES-1, and BEL-1 types. The SFO-1 *β*-lactamase was first reported in 1988 in a clinical *E. cloacae* isolate in Japan, and it confers resistance to third-generation cephalosporins (Matsumoto & Inoue, 1999); VEB was reported in China during an outbreak of infection caused by *E. cloacae* [31]. No document has yet reported the identification of the *bla*_Z_ gene that encodes class A enzymes in *E. cloacae*, and the *bla*_Z_ gene has been found only once in *Staphylococcus aureus* [16]. In this work, however, we isolated two *E. cloacae* strains (CG3 and CG4) that carried the *bla*_Z_ gene.

In addition to the *bla*_MIR_ and *bl*a_DHA_ genes identified in this work, genes encoding AmpC enzymes belonging to Ambler class C and Bush-Jacoby group 1 include *bla*_CMY_, *bla*_FOX_, *bla*_LAT_, *bla*_ACT_, *bla*_MOX_, *bla*_ACC_, and *bla*_BIL_ and their derivatives (H. [32]). The emergence of AmpC-producing *Enterobacter* spp. has been observed globally in health care-associated settings and in the community [33]. However, unlike most of the AmpC genes, *bla*_MIR_ has been found only in some strains of several *Enterobacter* spp., mainly in strains of *E. cloacae*. A total of 24 *bla*_MIR_ nucleotide sequences (between the *bla*_MIR-1_ and *bla*_MIR-21_ subtypes) are available in the NCBI nucleotide database; these sequences mainly came from the *ECC*, such as *E. cloacae* and *E. aerogenes*, as well as strains of *K. pneumoniae* and *E. coli*. In this work, 6 MIRs belonged 5 subtypes, including *bla*_MIR-3_, *bla*_MIR-5_, *bla*_MIR-17_, *bla*_MIR-21_, and *bla*_MIR-20_. Although the *bla*_MIR_ gene has been primarily identified on bacterial chromosomes, it is also encoded on plasmids. The AmpC *β*-lactamase *bla*_MIR-1_ was first described in *K. pneumoniae* plasmids [34]; *bla*_MIR-1_ confers resistance to penicillins and broad-spectrum cephalosporins, including cefoxitin and ceftibuten, but not to cefepime, cefpirome, meropenem, or imipenem. The resistance features of *bla*_MIR_ genes in human and animal isolates were different from those of some plasmid-encoded AmpC-type *β*-lactamase genes, such as *bla*_DHA_ and *bla*_CMY_, and have been reported worldwide to hydrolyze third-generation cephalosporins [35, 36]. The *bla*_MIR_ gene identified in this work was encoded on the chromosome and showed high sequence identity with other homologous *bla*_MIR_ genes found in other *Enterobacteriaceae*. Like the other previously reported MIRs, they showed resistance to ampicillin, cefazolin, cefmenoxime, and cefoxitin, but sensitive to fourth-generation cephalosporins (cefoselis) and monobactam (aztreonam).

To adapt to environmental changes, bacteria often harbor genes conferring resistance to toxic metal compounds; these genes include those encoding copper and arsenic ion transportation systems [37]. Copper sulfate is a common feed supplement for pigs, chickens, and calves worldwide. The copper-binding operon system (*PcoBCDE* and *CusR/S*), which is known to transport copper-derived compounds out of the bacterial cell to balance the concentration of copper salts, was elaborated on the pRJ1004 plasmid of *E. coli* isolates from piggeries in which the animals were provided food supplemented with copper sulfate [38]. Despite the identity of arsenic and arsenite compounds as high-toxicity compounds that are neither used in agriculture nor found in either the community or the hospital sector, the presence of arsenic resistance determinants on *Enterobacteriaceae* plasmids, especially the IncH-type plasmids, has been described before [9]. Three prototypes of *ars* operons, including the three-, four-, and five-gene arsenic resistance determinants, namely, *arsABC*, *arsABCD*, and *arsABCDR*, respectively, have been well documented, although novel resistance mechanisms have also been described [39]. The *arsABCDR* operon is related to resistance to arsenic-derived compounds, including arsine, arsenic, arsenite, and arsenate. Heavy metal resistance genes or gene clusters have been widely identified in different genera of both gram-positive and gram-negative bacteria and are encoded on both chromosomes and plasmids. In this work, on the plasmid pY546, we found four clusters of genes conferring resistance to heavy metals, such as arsenic (*arsABCDR*), tetrathionate (*ttrABCDRS*), and tellurite (*terCDEF*) as well as an incomplete copper resistance operon (*pcoBCDE/cusRS*). We must expect that bacteria have adapted to heavy metals with an increasing frequency.

## 5. Conclusion

In this work, through high-throughput sequencing, we identified twelve *β*-lactamase genotypes in 212 clinical *E. cloacae* isolates; of these, *bla*_Z_ has not yet been reported in *E. cloacae*. Furthermore, whole genome analysis of the *bla*_MIR_-carrying *E. cloacae* strain Y546 demonstrated that the strain harbored a large plasmid carrying a variety of gene clusters and genes, such as heavy metal resistance gene clusters (e.g., the *pco*, *ars*, *ter*, and *ttr* operons), conferring resistance to antimicrobials. Comparative genomics analysis showed that the sequences sharing the highest similarity to pY546 were plasmids from *K. pneumoniae* strains (44-49% similarity) and the chromosome of *E. coli* (40-41% similarity) and that the sequence fragments with the highest similarity to heavy metal resistance gene clusters on pY546 were from other plasmids and other chromosome sequences. The colocalization of antibiotic resistance genes and heavy metal resistance genes in the genomes of clinical pathogens, which may facilitate the persistence, coselection, and dissemination of these genes between different bacterial species or genera, is alarming and needs further surveillance.

## Figures and Tables

**Table 1:** Bacteria and plasmids used in this study.

Strain	Relevant characteristic(s)	Reference or source
BL21	*E. coli* BL21 was used as a host for the cloned *bla*_MIR_ gene	[14]
ATCC25922	*E. coli* ATCC25922 is FDA clinical isolate	[14]
CG34	*bla*_MIR_-producing isolate of *E. cloacae* CG34	This study
CG76	*bla*_MIR_-producing isolate of *E. cloacae* CG76	This study
CG85	*bla*_MIR_-producing isolate of *E. cloacae* CG85	This study
Y546	*bla*_MIR_-producing isolate of *E. cloacae* Y546	This study
Y482	*bla*_MIR_-producing isolate of *E. cloacae* Y482	This study
Y490	*bla*_MIR_-producing isolate of *E. cloacae* Y490	This study
pET28a/BL21	BL21 carrying expression vector pET28a, km^r^	This study
pET28a-*bla*_MIR-CG34_/BL21	BL21 carrying recombinant plasmid pET28a-*bla*_MIR-CG34_	This study
pET28a-*bla*_MIR-Y490_/BL21	BL21 carrying recombinant plasmid pET28a-*bla*_MIR-Y490_	This study
pET28a-*bla*_MIR-CG76_/BL21	BL21 carrying recombinant plasmid pET28a*-bla*_MIR-CG76_	This study
pET28a-*bla*_MIR-Y546_/BL21	BL21 carrying recombinant plasmid pET28a-*bla*_MIR-Y546_	This study
pET28a-*bla*_MIR-CG85_/BL21	BL21 carrying recombinant plasmid pET28a-*bla*_MIR-CG85_	This study
pET28a-*bla*_MIR-Y482_/BL21	BL21 carrying recombinant plasmid pET28a-*bla*_MIR-Y482_	This study
pET28a-*bla*_CTX-M-9a-Y546_/BL21	BL21 carrying recombinant plasmid pET28a-*bla*_CTX-M-9a-Y546_	This study
pET28a-*bla*_SHV-12-Y546_/BL21	BL21 carrying recombinant plasmid pET28a-bla_SHV-12-Y546_	This study

km: kanamycin; ^r^: resistant.

**Table 2:** Primers used in the study for the detection of *β*-lactamase-encoding genes.

Primer	Target(s)	Sequence (5′-3′)	Annealing temperature (°C)	Amplicon size (bp)	Reference
veb-sf	*bla*_VEB_	GATTGCTTTAGCCGTTTTGTC	50	452	[15]
veb-sr		ATCGGTTACTTCCTGTTGTTGTTTC			
z-sf	*bla*_Z_	ACAGTTCACATGCCAAAGAGT	50	479	[16]
z-sr		CTTACCGAAAGCAGCAGGTG			
mir-sf	*bla*_MIR_	GCCGCACCGATGTCCGAAAAA	50	545	[18]
mir-sr		GGTTTAAAGACCCGCGTCGTCATGG			
azecl-29-sf	*bla*_AZECL-29_	GTCTTTACGCTAACACCAGCATCGG	50	381	[17]
azecl-29-sr		TCAGCATTTCCCAGCCCAATC			
veb-ff	*bla*_VEB_	*CGGGATCC*ATGAAAATCGTAAAAAGGATATTAT	50	918	This study
veb-fr		*CCCAAGCT*TTATTTATTCAAATAGTAATTCCACG			
z-ff	*bla*_Z_	*CGGGATCC*ATGAAAAAGTTAATATTTTTAATTG	50	864	This study
z-fr		*CCCAAGCT*TTAAAATTCCTTCATTACACTCTTG			
mir-ff	*bla*_MIR_	*CGGGATCC*ATGATGACAAAATCCCTAAGCTGTG	66	1164	This study
mir-fr		*CCCAAGCT*TTACTGCAGCGCGTCGAGGATACGG			
azecl-29-ff	*bla*_AZECL-29_	*CGGAATTC*ATGATGAAAAAAAACCTAAGCTGTG	60	1164	This study
azecl-29-fr		*CCCAAGCT*TTACTGCAGCGCGTCGAGGATACG			
ctx-m-9a-ff	*bla*_CTX-M-9a_	*CGGGATCC*ATGGTGACAAAGAGAGTGCAACGGA	60	876	This study
ctx-m-9a-fr		*CCAAGCTT*TTACAGCCCTTCGGCGATGATTCTC			
shv-12-ff	*bla*_SHV-12_	*CGGGATCC*GTGGTTATGCGTTATATTCGCCTGT	60	867	This study
shv-12-fr		*CCAAGCTT*TTAGCGTTGCCAGTGCTCGATCAGC			

Underlined sequences denote restriction endonuclease sites. sf: forward screening primer; sr: reverse screening primer; ff: forward full-length primer; fr: reverse full-length primer.

**Table 3:** The MIC values of antibacterial drugs for the strains (*μ*g/mL).

Strains	AMP	KAN	CAZ	CTX	CPZ	CMN	CEF	CFZ	FOX	CRO	ATM	PRL	PTZ	IMC
ATCC25922	4	2	0.25	0.0625	0.125	1	0.125	2	4	0.0313	0.125	2	4	0.25
BL21	0.5	4	0.0625	0.0156	0.0156	1	0.0313	2	2	0.0156	0.0156	0.5	0.5	0.5
pET-28a/BL21^∗^	0.5	32	0.0625	0.0156	0.0156	1	0.0313	2	2	0.0156	0.0156	0.5	0.5	0.5
CG34	256	0.5	0.125	0.125	0.0625	1024	0.0625	512	1024	0.0313	0.25	2	2	4
Y490	256	2	0.5	0.25	1	>1024	0.125	512	1024	0.5	0.0313	2	4	2
CG76	>1024	8	128	128	64	1024	16	>1024	1024	512	32	512	32	8
Y546	512	2	2	1	2	>1024	0.25	1024	1024	2	1	8	8	4
CG85	>1024	4	1	1	1	>1204	0.25	1024	>1024	1	0.25	4	4	4
Y482	64	2	0.25	0.125	0.25	1024	256	512	0.0625	0	0.125	2	2	0.5
pET-28a-*bla*_MIR_ (CG34)/BL21^∗^	128	64	4	4	1	32	0.125	256	32	4	1	8	8	1
pET-28a-*bla*_MIR_ (Y490)/BL21^∗^	128	64	4	4	0.5	32	0.0625	256	32	4	1	8	4	1
pET-28a-*bla*_MIR_ (CG76)/BL21^∗^	128	64	4	4	1	32	0.0313	256	32	4	1	8	4	1
pET-28a-*bla*_MIR_ (Y546)/BL21^∗^	128	64	4	4	1	32	0.0625	256	32	4	1	8	8	1
pET-28a-*bla*_MIR_ (CG85)/BL21^∗^	128	64	4	4	1	32	0.125	256	32	4	1	8	8	1
pET-28a-*bla*_MIR_ (Y482)/BL21^∗^	128	64	4	4	1	32	0.125	256	32	4	1	8	8	1
pET-28a-*bla*_CTX-M-9a_/BL21^∗^	8	64	0.25	4	1	4	0.03	64	4	0.03	0.03	2	1	1
pET-28a-*bla*_SHV-12_/BL21^∗^	8	64	2	4	1	4	0.25	64	4	0.03	0.03	32	2	2

^∗^IPTG was added to a final concentration of 1 mmol/L to standard Mueller-Hinton (M-H) agar plates. All MIC determinations were performed by agar dilution assays. FOX: cefoxitin; CAZ: ceftazidime; CTX: cefotaxime; AMP: ampicillin; KAN: kanamycin; CPZ: cefoperazone; CMN: cefminox; CEF: cefoselis; CFZ: cefazolin; CRO: ceftriaxone; ATM: aztreonam; PRL: piperacillin; PTZ: piperacillin/tazobactam; IMC: imipenem/cilastatin sodium hydrate.

**Table 4:** General features of the Y546 genome.

	Chromosome	Plasmid
Size (bp)	4,787,919	208,741
GC content (%)	56.02	52.63
ORFs	4312	234
Known proteins	3476 (80.6%)	137 (58.5%)
Hypothetical proteins	836 (19.4%)	97 (41.5%)
Protein coding (%)	88.02	82.80
Average ORF length (bp)	977	738
Average protein length (aa)	324	245
tRNAs	85	0
rRNA operons	(16S-23S-5S) ^∗^7	0
	16S-23S-5S-5S	

## Data Availability

The data used to support the findings of this study are included within the article.

## Additional Points

*Highlights*. Twelve *β*-lactamase genotypes, including *bla*_SHV_, *bla*_TEM_, *bla*_DHA_, *bla*_CTX-M_, *bla*_Z_, *bla*_VEB_, *bla*_KLUC_, *bla*_MIR_, *bla*_SFO_, *bla*_AZECL-29_, *bla*_OXA_, and *bla*_ACT_, were identified in 212 *E. cloacae* genomes. *bla*_Z_ was found in *E. cloacae* for the first time. *bla*_MIR_ was identified in six *E. cloacae* strains that were not clonally related. The gene clusters related to resistance to heavy metals (such as copper, arsenic, and tellurite) were identified to be encoded on the pY546 plasmid. The sequences with the highest similarity to pY546 were on plasmids from *K. pneumoniae* strains and on *E. coli* chromosomes; in addition, the sequence fragments with the highest similarity to the heavy metal resistance gene clusters on pY546 were from other sources.

## Supplementary Materials

Supplementary Materials Table S1: the reference *β*-lactamase gene sequences collected from GenBank. Table S2: the mapping result of *β*-lactamase resistance genes in the *E. cloacae* pooled genomic sequences. Table S3: annotation result of the pY546 genome.
