# Supplementary material for: The β-Lactamase Gene Profile and a Plasmid-Carrying Multiple Heavy Metal Resistance Genes of Enterobacter cloacae
Source: Int J Genomics. 2018 Dec 20;2018:4989602. doi: 10.1155/2018/4989602 (PMC6317114; doi:10.1155/2018/4989602)
Supplement: Supplementary Materials — Table S1: the reference β-lactamase gene sequences collected from GenBank. Table S2: the mapping result of β-lactamase resistance genes in the E. cloacae pooled genomic sequences. Table S3: annotation result of the pY546 genome. [file 4989602.f1.doc]

Table S1: The reference β-lactamase gene sequences collected from the GenBank.

| Amber class A | accession number | Amber class B | accession number | Amber class C | accession number | Amber class D | accession number |
| --- | --- | --- | --- | --- | --- | --- | --- |
| *bla*_ACI_ | AJ007350.1 | *bla*_B_ | CP003788.1 | *bla*_ACC_ | EU650653.1 | *amp*_H_ | CP000647.1 |
| *bla*_AER_ | U14748.1 | *bla*_CGB_ | EF672680.1 | *bla*_ACTm_ | AM076977.1 | *amp*S | CP003235.1 |
| *bla*_AST_ | AF279904.1 | *bla*_DIM_ | KC004136.2 | *bla*_BIL_ | X74512.1 | *bla*_LCR_ | CP002878.1 |
| *bla*_BEL_ | FJ666063.1 | *bla*_EBR_ | AF416700.1 | *bla*_BUT_ | AJ415568.1 | *bla*_NPS_ | NG_035412.1 |
| *bla*_BES_ | NG_035230.1 | *bla*_GIM_ | KT440883.1 | *bla*_CFE_ | NG_035840.1 | *bla*_OXA_ | KT988020.1 |
| *bla*_BIC_ | GQ260093.1 | *bla*_GOB_ | DQ004496.1 | *bla*_CMG_ | AY265892.1 |  |  |
| *bla*_BPS_ | CP012093.1 | *bla*_IMP_ | AY055216.1 | *bla*_CMY_ | AF373217.2 |  |  |
| *bla*_CARB_ | GU188441.1 | *bla*_IND_ | AB529520.1 | *bla*_DHA_ | JX495964.1 |  |  |
| *bla*_CKO_ | AF477396.1 | *bla*_JO_HN | AY028464.1 | *bla*_FOX_ | AJ703795.1 |  |  |
| *bla*_CTX-M_ | NG_039596.1 | *bla*_MUS_ | AF441286.1 | *bla*_LEN_ | AJ635425.1 |  |  |
| *bla*_DES_ | AF426161.1 | *bla*_NDM_ | NG_041520.1 | *bla*_MIR_ | FJ237367.1 |  |  |
| *bla*_ERP_ | AY077733.1 | *bla*_SPM_ | GU831554.1 | *bla*_OCH_ | AJ295342.1 |  |  |
| *bla*_FAR_ | AF024601.1 | *bla*_TUS_ | AF441287.1 | *bla*_OKP-A_ | FJ534513.1 |  |  |
| *bla*_GES_ | KT984195.1 | *bla*_VIM_ | JX185132.1 | *bla*_OXY_ | JX559323.1 |  |  |
| *bla*_IMI_ | DQ173429.1 | *cep*A | FR688019.1 | *bla*_TRU_ | EU046614.1 |  |  |
| *bla*_KLUA_ | KR827036.1 | *cfi*A | AY372696.1 | *bla*_ZEG_ | AY265891.1 |  |  |
| *bla*_KLUC_ | NG_041577.1 | *imi*S | Y10415.1 | *cep*H | AJ276030.1 |  |  |
| *bla*_KPC_ | KR014106.1 |  |  |  |  |  |  |
| *bla*_LUT_ | CP002085.1 |  |  |  |  |  |  |
| *bla*_PER_ | CP010351.1 |  |  |  |  |  |  |
| *bla*_PME_ | HQ541434.2 |  |  |  |  |  |  |
| *bla*_RAHN_ | HM114350.1 |  |  |  |  |  |  |
| *bla*_ROB_ | KJ910983.1 |  |  |  |  |  |  |
| *bla*_SED_ | AF321608.1 |  |  |  |  |  |  |
| *bla*_SFC_ | AY354402.1 |  |  |  |  |  |  |
| *bla*_SFO_ | NG_035150.1 |  |  |  |  |  |  |
| *bla*_SHV_ | KF585134.1 |  |  |  |  |  |  |
| *bla*_SME_ | JF974075.1 |  |  |  |  |  |  |
| *bla*_TEM_ | AY072920.1 |  |  |  |  |  |  |
| *bla*_TLA_ | NG_040885.1 |  |  |  |  |  |  |
| *bla*_TOHO_ | AY671905.1 |  |  |  |  |  |  |
| *bla*_VEB_ | LC107425.1 |  |  |  |  |  |  |
| *bla*_Z_ | FN435327.1 |  |  |  |  |  |  |
| *cdi*A | CP009166.1 |  |  |  |  |  |  |
| *cfx*A | AF118110.1 |  |  |  |  |  |  |
| *cum*A | X80128.1 |  |  |  |  |  |  |

Table S2: The mapping result of β-lactamase resistance genes in the *E. cloacae* pooled genomic sequences.

|  | Genotype |  | Coverage^a^ |  | Abundance^b^ |  |
| --- | --- | --- | --- | --- | --- | --- |
|  | *bla*_VEB_ |  | 1 |  | 6.54 |  |
|  | *bla*_Z_ |  | 1 |  | 1.89 |  |
|  | *bla*_DHA_ |  | 1 |  | 191.12 |  |
|  | *bla*_SHV_ |  | 1 |  | 226.77 |  |
|  | *bla*_MIR_ |  | 1 |  | 10.89 |  |
|  | *bla*_TEM_ |  | 1 |  | 466.15 |  |
|  | *bla*_OXA_ |  | 1 |  | 14.92 |  |
|  | *bla*_CTX-M-9_ |  | 1 |  | 141.73 |  |
|  | *bla*_SFO_ |  | 1 |  | 11.47 |  |
|  | *amp*_H_ |  | 1 |  | 9.69 |  |
|  | *bla*_CTX-M-1_ |  | 1 |  | 140.66 |  |
|  | *bla*_KLUC_ |  | 1 |  | 10.52 |  |
|  | *bla*_ACT_ |  | 1 |  | 32.23 |  |
|  | *bla*_CMG_ |  | 1 |  | 14.05 |  |

^a^ The ratio of the reference sequence covered by the sequencing reads to the full length of the reference sequence.

^b^ The relative abundance (sequencing depth) for a certain gene was calculated through the cumulative nucleotide length of the mapped reads on the gene divided by the gene size.

Table S3: Annotation result of the pY546 genome.

| ORFs | Gene | Position (bp) | Length (aa^*^) | Description | Accession no. |
| --- | --- | --- | --- | --- | --- |
| pY546-001 | - | 16-540 | 174 | Uncharacterized protein | SAW11614.1 |
| pY546-002 | - | 713-2,335 | 540 | MULTISPECIES: hypothetical protein | WP_032174388.1 |
| pY546-003 | - | 3,035-2,328 | 235 | conserved hypothetical protein | ZP_07132914.1 |
| pY546-004 | - | 3,830-3,084 | 248 | hypothetical protein | EWD70847.1 |
| pY546-005 | - | 5,441-3,867 | 524 | Transporter | OKN15065.1 |
| pY546-006 | - | 7,521-5,434 | 695 | hypothetical protein P821_05414 | EWD70845.1 |
| pY546-007 | *tns*A | 8,335-7,508 | 275 | TnsA | WP_032731983.1 |
| pY546-008 | - | 8,432-9,370 | 312 | Pyridine nucleotide-disulfide oxidoreductase | WP_001046322.1 |
| pY546-009 | *IS*1 | 9,847-10,176 | 109 | *IS*1 family transposase (Fragment) | ATZ33656.1 |
| pY546-010 | - | 10,394-10,732 | 112 | Uncharacterized protein | WP_004150624.1 |
| pY546-011 | - | 11,089-10,829 | 86 | DUF2534 domain-containing protein | WP_023313931.1 |
| pY546-012 | - | 11,871-11,146 | 241 | TonB-dependent copper receptor | YP_050936.1 |
| pY546-013 | - | 12,860-11,871 | 329 | TonB-dependent copper receptor | WP_064142075.1 |
| pY546-014 | - | 13,207-12,812 | 131 | TonB-dependent copper receptor | WP_064142075.1 |
| pY546-015 | - | 13,592-13,290 | 100 | hypothetical protein L401_05076 | ESM32079.1 |
| pY546-016 | *IS*110 | 14,070-15,074 | 334 | *IS*110 family transposase | WP_040120329.1 |
| pY546-017 | - | 15,699-15,421 | 92 | Uncharacterized protein | WP_004118062.1 |
| pY546-018 | - | 16,005-15,793 | 70 | Uncharacterized protein | WP_004181742.1 |
| pY546-019 | *yad*A | 17,305-16,913 | 130 | YadA | EMH91336.1 |
| pY546-020 | *rep*B | 18,622-19,506 | 294 | RepB | WP_004210308.1 |
| pY546-021 | - | 19,667-20,581 | 304 | Uncharacterized protein | SAY21609.1 |
| pY546-022 | *IS*110 | 21,050-22,075 | 341 | *IS*110 family transposase | WP_001101446.1 |
| pY546-023 | *IS*5/*IS*1182 | 22,370-23,338 | 322 | *IS*5/*IS*1182 family transposase | WP_074170039.1 |
| pY546-024 | - | 23,491-24,027 | 178 | Plasmid transfer protein | WP_080876707.1 |
| pY546-025 | - | 24,886-25,254 | 122 | hypothetical protein L458_05084 | ESL57879.1 |
| pY546-026 | - | 25,239-25,526 | 95 | Uncharacterized protein | ESL57878.1 |
| pY546-027 | - | 25,957-26,112 | 51 | Uncharacterized protein | WP_011154519.1 |
| pY546-028 | *rep*A | 27,358-26,348 | 336 | DNA replication protein RepA | ESL46625.1 |
| pY546-029 | *orfA* | 27,748-28,011 | 87 | *IS*3 family transposase orfA (plasmid) | YP_003602620.1 |
| pY546-030 | *IS*3 | 28,038-28,868 | 276 | *IS*3 family transposase | WP_088251124.1 |
| pY546-031 | *sopA* | 29,313-30,479 | 388 | MULT*IS*PECIES: protein SopA | WP_023287153.1 |
| pY546-032 | *sop*B | 30,479-31,450 | 323 | SopB | AAR07880.1 |
| pY546-033 | *sam*B | 33,055-32,909 | 48 | Protein SamB (fragment) | CEL88990.1 |
| pY546-034 | - | 33,354-33,728 | 124 | Transposase | WP_040027444.1 |
| pY546-035 | *IS*66 | 33,725-34,075 | 116 | *IS*66 family insertion sequence | WP_004189161.1 |
| pY546-036 | *IS*Kox1 | 34,294-35,640 | 448 | *IS*66-like element *IS*Kox1 family transposase | WP_087879639.1 |
| pY546-037 | *IS*110 | 36,860-35,892 | 322 | *IS*110 family transposase | WP_011154513.1 |
| pY546-038 | *ins*A | 37,076-37,240 | 54 | *IS*2 insertion element repressor *Ins*A; KpLE2 phage-like element (fragment) | CDI15648.1 |
| pY546-039 | *ins*D | 37,288-38,103 | 271 | Transposase *Ins*D for insertion element *IS*2A/D/F/H/I/K | EKB82436.1 |
| pY546-040 | - | 38,124-38,543 | 139 | Uncharacterized protein | SAQ66961.1 |
| pY546-041 | - | 38,576-38,983 | 135 | MULTISPECIES: hypothetical protein | WP_004186937.1 |
| pY546-042 | - | 39,026-39,985 | 319 | MULTISPECIES: hypothetical protein | WP_011154511.1 |
| pY546-043 | - | 39,982-40,740 | 252 | MULTISPECIES: hypothetical protein | WP_063938344.1 |
| pY546-044 | - | 40,737-41,060 | 107 | hypothetical protein | WP_049256912.1 |
| pY546-045 | - | 41,212-41,529 | 105 | MULTISPECIES: hypothetical protein | WP_004213829.1 |
| pY546-046 | - | 42,731-41,595 | 378 | MULTISPECIES: recombinase | WP_004213833.1 |
| pY546-047 | - | 42,909-43,163 | 84 | MULTISPECIES: hypothetical protein | WP_004213836.1 |
| pY546-048 | - | 43,249-44,172 | 307 | MULTISPECIES: hypothetical protein | WP_016946353.1 |
| pY546-049 | *IS*5/*IS*1182 | 45,186-44,218 | 322 | *IS*5/*IS*1182 family transposase | WP_014839937.1 |
| pY546-050 | △*ins*D | 45,261-45,776 | 171 | transposase *Ins*D for insertion element *IS*2A/D/F/H/I/K | KDL41500.1 |
| pY546-051 | - | 45,909-46,220 | 103 | Cytoplasmic protein | WP_079899846.1 |
| pY546-052 | - | 46,217-46,339 | 40 | hypothetical protein pK2044_00510 | YP_001687932.1 |
| pY546-053 | - | 46,318-46,635 | 105 | XRE family transcriptional regulator | WP_011251286.1 |
| pY546-054 | *IS*5 | 46,782-47,750 | 322 | *IS*5 family transposase | WP_074194776.1 |
| pY546-055 | - | 48,702-49,109 | 135 | hypothetical protein | WP_085802832.1 |
| pY546-056 | *IS*66 | 49,106-49,363 | 85 | MULTISPECIES: *IS*66 family insertion sequence hypothetical protein | WP_032414478.1 |
| pY546-057 | △*IS*5/*IS*1182 | 49,823-49,386 | 145 | *IS*5/*IS*1182 family transposase, partial | PPJ81848.1 |
| pY546-058 | - | 50,190-49,825 | 121 | hypothetical protein L458_04938 | ESL58126.1 |
| pY546-059 | - | 50,992-50,204 | 262 | MULTISPECIES: hypothetical protein | WP_040217257.1 |
| pY546-060 | - | 51,456-51,013 | 147 | MULTISPECIES: hypothetical protein | WP_004181916.1 |
| pY546-061 | - | 52,052-52,621 | 189 | Uncharacterized protein | SLQ83725.1 |
| pY546-062 | *IS*5/*IS*1182 | 53,633-54,601 | 322 | *IS*5/*IS*1182 family transposase | WP_088717386.1 |
| pY546-063 | - | 54,948-55,328 | 126 | Transposase | EGB40077.1 |
| pY546-064 | *IS*66 | 55,325-55,672 | 115 | *IS*66 family insertion sequence hypothetical protein | WP_096937460.1 |
| pY546-065 | *IS*Ec8 | 55,722-57,260 | 512 | *IS*66-like element *IS*Ec8 family transposase | WP_001547431.1 |
| pY546-066 | - | 57,773-57,363 | 136 | putative membrane protein | ZP_05967700.1 |
| pY546-067 | *IS*5/*IS*1182 | 58,410-57,751 | 219 | *IS*5/*IS*1182 family transposase, partial | OVU24874.1 |
| pY546-068 | - | 59,370-58,588 | 260 | resolvase | YP_003754077.1 |
| pY546-069 | - | 60,257-59,367 | 296 | hypothetical protein SM87_06121 | KMI22609.1 |
| pY546-070 | - | 60,558-60,229 | 109 | MULTISPECIES: hypothetical protein | WP_048268852.1 |
| pY546-071 | *pif*C | 61,117-62,205 | 362 | transcriptional repressor PifC | ZP_08361286.1 |
| pY546-072 | - | 62,216-63,589 | 457 | hypothetical protein | WP_086528282.1 |
| pY546-073 | - | 63,774-65,102 | 442 | nucleotidyltransferase | WP_004206607.1 |
| pY546-074 | - | 65,110-65,685 | 191 | MULTISPECIES: hypothetical protein | WP_004206608.1 |
| pY546-075 | - | 66,107-67,057 | 316 | conserved hypothetical protein | ZP_08386534.1 |
| pY546-076 | - | 67,350-68,264 | 304 | hypothetical protein | WP_052686739.1 |
| pY546-077 | *IS*5/*IS*1182 | 69,523-68,555 | 322 | *IS*5/*IS*1182 family transposase | WP_016809156.1 |
| pY546-078 | - | 69,616-69,813 | 65 | hypothetical protein | EFU56400.1 |
| pY546-079 | - | 70,100-69,810 | 96 | MULTISPECIES: hypothetical protein | WP_001535717.1 |
| pY546-080 | - | 71,230-70,100 | 376 | conserved hypothetical protein | EFU56398.1 |
| pY546-081 | - | 72,345-71,260 | 361 | conserved domain protein | EFU56397.1 |
| pY546-082 | - | 72,693-72,956 | 87 | hypothetical protein | WP_087661548.1 |
| pY546-083 | - | 72,953-73,519 | 188 | hypothetical protein | WP_087661549.1 |
| pY546-084 | - | 73,550-74,044 | 164 | DNA-binding protein | WP_087661550.1 |
| pY546-085 | - | 74,094-74,297 | 67 | haemolysin expression modulating protein | YP_002235789.1 |
| pY546-086 | - | 74,311-74,475 | 54 | hypothetical protein, partial | WP_087661551.1 |
| pY546-087 | - | 74,493-74,885 | 130 | *IS*5/*IS*1182 family transposase, partial | OVT64629.1 |
| pY546-088 | - | 74,963-75,367 | 134 | hypothetical protein pK29_p251 | YP_001965921.1 |
| pY546-089 | *IS*66 | 75,364-75,711 | 115 | MULTISPECIES: *IS*66 family insertion sequence hypothetical protein | WP_000612626.1 |
| pY546-090 | *IS*Kpn24 | 75,760-77,298 | 512 | *IS*66-like element *IS*Kpn24 family transposase | WP_023316587.1 |
| pY546-091 | △*pco*B | 77,862-77,641 | 73 | copper resistance protein CopB, partial | PLP50098.1 |
| pY546-092 | *pco*C | 77,941-78,321 | 126 | copper resistance system chaperone PcoC | WP_000025662.1 |
| pY546-093 | *pco*D | 78,326-79,255 | 309 | copper resistance protein CopD | WP_063840952.1 |
| pY546-094 | *cus*R | 79,310-79,990 | 226 | transcriptional regulatory protein PcoR (plasmid) | NP_943472.1 |
| pY546-095 | *cus*S | 79,987-81,387 | 466 | sensor histidine kinase | WP_001211180.1 |
| pY546-096 | *pco*E | 81,605-82,018 | 137 | copper-binding protein | WP_000723069.1 |
| pY546-097 | *tnp*A | 83,637-82,048 | 529 | *Tnp*A (plasmid) | ADL13996.1 |
| pY546-098 | *IS*66 | 84,017-83,667 | 116 | *IS*66 family insertion sequence hypothetical protein | WP_032414478.1 |
| pY546-099 | - | 84,421-84,014 | 135 | MULTISPECIES: hypothetical protein | WP_015632445.1 |
| pY546-100 | *ars*C | 85,048-84,623 | 141 | ArsC (plasmid) | YP_001965822.1 |
| pY546-101 | *ars*B | 86,350-85,061 | 429 | arsenic efflux pump protein ArsB | ZP_08303599.1 |
| pY546-102 | *ars*A | 88,149-86,398 | 583 | arsenical pump-driving ATPase | YP_001338529.1 |
| pY546-103 | *ars*D | 88,528-88,208 | 106 | arsenical resistance operon transcriptional repressor ArsD | PXH03734.1 |
| pY546-104 | *ars*R | 88,928-88,578 | 116 | Arsenical resistance operon repressor | CAQ90297.1 |
| pY546-105 | *ret*A | 90,70-689,219 | 495 | putative *Ret*A reverse transcriptase | YP_001966181.1 |
| pY546-106 | *umu*D | 90,800-91,207 | 135 | Error-prone repair protein UmuD | AJD77245.1 |
| pY546-107 | *ke*F | 91,553-92,752 | 399 | Potassium transporter Kef | OGS71459.1 |
| pY546-108 | *usp*A | 92,804-93,223 | 139 | Universal stress protein UspA | AKE62345.1 |
| pY546-109 | *mut*S | 93,462-95,000 | 512 | DNA mismatch repair protein MutS | WP_046499031.1 |
| pY546-110 | *mut*S | 95,569-96,516 | 315 | DNA mismatch repair protein MutS | WP_046499035.1 |
| pY546-111 | - | 97,794-96,607 | 395 | divalent cation transporter (plasmid) | AKE62346.1 |
| pY546-112 | - | 98,918-97,740 | 392 | divalent cation transporter | WP_052747017.1 |
| pY546-113 | *crc*B | 99,402-99,028 | 124 | Putative fluoride ion transporter CrcB | WP_016154442.1 |
| pY546-114 | - | 99,919-99,437 | 160 | Phosphoglycerate mutase 1 family | EJU32529.1 |
| pY546-115 | - | 100,253-100,444 | 63 | MULTISPECIES: hypothetical protein | WP_009652435.1 |
| pY546-116 | - | 101,736-100,450 | 428 | phosphopyruvate hydratase | WP_009652415.1 |
| pY546-117 | - | 102,180-101,752 | 142 | universal stress protein | WP_009652460.1 |
| pY546-118 | - | 103,161-102,184 | 325 | MULTISPECIES: pyrophosphatase | WP_009652407.1 |
| pY546-119 | - | 103,478-104,056 | 192 | undecaprenyl-diphosphatase | WP_046499050.1 |
| pY546-120 | - | 104,415-105,089 | 224 | Voltage-gated chloride channel protein | WP_046499056.1 |
| pY546-121 | - | 105,086-105,787 | 233 | Voltage-gated chloride channel protein | WP_046499056.1 |
| pY546-122 | - | 105,784-106,173 | 129 | MULTISPECIES: hypothetical protein | WP_023223701.1 |
| pY546-123 | - | 106,512-107,291 | 259 | HAD family hydrolase | WP_046499061.1 |
| pY546-124 | - | 107,478-107,915 | 145 | Type III restriction enzyme, res subunit | CEL85066.1 |
| pY546-125 | - | 108,110-108,724 | 204 | Type III restriction enzyme, res subunit | CEL85066.1 |
| pY546-126 | - | 108,848-109,036 | 62 | hypothetical protein | WP_044865706.1 |
| pY546-127 | *orfA* | 109,086-109,349 | 87 | putative *IS*3 family transposase orfA | YP_003602620.1 |
| pY546-128 | *IS*3 | 109,376-109,999 | 207 | *IS*3 family transposase | WP_064757125.1 |
| pY546-129 | - | 110,543-111,304 | 253 | hypothetical protein | WP_080950113.1 |
| pY546-130 | - | 112,850-111,435 | 471 | hypothetical protein | WP_046499071.1 |
| pY546-131 | - | 114,574-112,847 | 575 | hypothetical protein | WP_046499074.1 |
| pY546-132 | *IS*Kpn26 | 115,768-114,869 | 299 | *IS*5-like element *IS*Kpn26 family transposase | WP_079848467.1 |
| pY546-133 | - | 116,839-115,895 | 314 | Phage integrase | ESG84989.1 |
| pY546-134 | - | 117,370-117,038 | 110 | hypothetical protein KP13_05807 | AHE47513.1 |
| pY546-135 | - | 117,827-117,429 | 132 | MULTISPECIES: DNA-binding protein | WP_004181717.1 |
| pY546-136 | - | 119,048-118,230 | 272 | DNA repair protein (plasmid) | ART08676.1 |
| pY546-137 | △*sop*B | 119,675-119,157 | 172 | SopB | AAR07880.1 |
| pY546-138 | - | 119,834-121,105 | 423 | hypothetical protein | WP_064164944.1 |
| pY546-139 | *ins*H | 122,114-123,130 | 338 | transposase *Ins*H for insertion sequence element *IS*5 | EGI12228.1 |
| pY546-140 | *IS*5/*IS*1182 | 123,314-124,330 | 338 | *IS*5/*IS*1182 family transposase | WP_077875138.1 |
| pY546-141 | - | 124,642-124,439 | 67 | MULTISPECIES: hypothetical protein | WP_000528119.1 |
| pY546-142 | *ttr*R | 125,319-124,735 | 194 | MULTISPECIES: two-component system response regulator TtrR | WP_000974596.1 |
| pY546-143 | *ttr*S | 126,976-125,294 | 560 | TtrS | EKK44618.1 |
| pY546-144 | *ttr*B | 127,255-127,989 | 244 | tetrathionate reductase subunit TtrB | WP_077910396.1 |
| pY546-145 | *ttr*C | 127,990-129,012 | 340 | tetrathionate reductase subunit TtrC | WP_044864557.1 |
| pY546-146 | *ttr*A | 129,005-132,070 | 1021 | tetrathionate reductase subunit TtrA | WP_000002334.1 |
| pY546-147 | - | 132,977-132,165 | 270 | methionine ABC transporter substrate-binding protein | WP_046499266.1 |
| pY546-148 | △*IS*5/*IS*1182 | 133,046-133,600 | 184 | *IS*5/*IS*1182 family transposase, partial | WP_077816045.1 |
| pY546-149 | *ins*H | 133,610-134,062 | 150 | transposase *Ins*H for insertion sequence element *IS*5 domain protein | EFU99656.1 |
| pY546-150 | - | 134,120-134,482 | 120 | MULTISPECIES: hypothetical protein | WP_004181725.1 |
| pY546-151 | - | 134,510-134,839 | 109 | MULTISPECIES: hypothetical protein | WP_053263858.1 |
| pY546-152 | - | 135,067-135,714 | 215 | haloacid dehalogenase | WP_042932546.1 |
| pY546-153 | *ter*F | 137,181-136,051 | 376 | tellurium resistance protein TerF | WP_080876677.1 |
| pY546-154 | *ter*E | 137,951-137,376 | 191 | tellurium resistance protein TerE | WP_000301247.1 |
| pY546-155 | *ter*D | 138,598-138,020 | 192 | tellurium resistance protein TerD | WP_000116680.1 |
| pY546-156 | *ter*C | 138,912-138,637 | 91 | Tellurium resistance protein TerC | CZV56809.1 |
| pY546-157 | *orfA* | 138,965-139,243 | 92 | putative *IS*3 family transposase orfA | YP_003602620.1 |
| pY546-158 | *IS*3 | 139,300-140,112 | 270 | *IS*3 family transposase | EWD74115.1 |
| pY546-159 | - | 141,405-140,428 | 325 | MULTISPECIES: hypothetical protein | WP_049131336.1 |
| pY546-160 | - | 143,340-141,409 | 643 | MULTISPECIES: hypothetical protein | WP_032731868.1 |
| pY546-161 | - | 143,707-143,342 | 121 | MULTISPECIES: hypothetical protein | WP_032731867.1 |
| pY546-162 | - | 145,545-143,707 | 612 | MULTISPECIES: hypothetical protein | WP_032731867.1 |
| pY546-163 | *orfB* | 146,748-145,996 | 250 | *IS*3 family element, transposase orfB | YP_002235668.1 |
| pY546-164 | - | 147,004-146,705 | 99 | transposase | EWF76382.1 |
| pY546-165 | - | 147,184-147,465 | 93 | putative prophage primase | YP_003466634.1 |
| pY546-166 | - | 147,609-147,998 | 129 | hypothetical protein | WP_077260294.1 |
| pY546-167 | - | 147,995-148,564 | 189 | Transposase | EWD70907.1 |
| pY546-168 | - | 149,176-150,246 | 356 | MULTISPECIES: hypothetical protein | WP_032732008.1 |
| pY546-169 | *IS*Spu2 | 150,878-150,516 | 120 | *IS*630-like element *IS*Spu2 family transposase | WP_101978035.1 |
| pY546-170 | - | 152,728-151,577 | 383 | conserved hypothetical protein | AEA84305.1 |
| pY546-171 | - | 153,718-152,753 | 321 | Zn-dependent protease | WP_032732006.1 |
| pY546-172 | - | 154,193-153,696 | 165 | conserved hypothetical protein | ZP_07136039.1 |
| pY546-173 | - | 155,905-154,190 | 571 | sodium:proton exchanger | WP_032732004.1 |
| pY546-174 | - | 156,349-155,909 | 146 | thiol reductase thioredoxin | WP_000786814.1 |
| pY546-175 | - | 157,484-156,339 | 381 | MULTISPECIES: hypothetical protein | WP_032732003.1 |
| pY546-176 | *hde*D | 158,175-157,564 | 203 | HdeD family acid-resistance protein | WP_032427177.1 |
| pY546-177 | - | 159,151-158,264 | 295 | hypothetical protein pC13298_p7 | YP_003864369.1 |
| pY546-178 | - | 160,168-159,254 | 304 | hypothetical protein pC13298_p6 | YP_003864368.1 |
| pY546-179 | - | 160,649-160,221 | 142 | hypothetical protein Bmul_2294 | YP_001580476.1 |
| pY546-180 | *fts*H | 161,966-160,737 | 409 | cell division protein FtsH | WP_032731999.1 |
| pY546-181 | *fts*H | 162,544-162,017 | 175 | FtsH Extracellular family protein | KDX28849.1 |
| pY546-182 | - | 162,796-162,605 | 63 | hypothetical protein | WP_044784781.1 |
| pY546-183 | *cl*P | 165,645-162,796 | 949 | ATP-dependent Clp protease ATP-binding subunit | WP_032731998.1 |
| pY546-184 | - | 166,320-165,751 | 189 | Hsp20/alpha crystallin family protein | WP_032731995.1 |
| pY546-185 | - | 166,636-166,355 | 93 | DNA-binding protein | WP_032732047.1 |
| pY546-186 | - | 167,141-166,890 | 83 | putative transposase | EFZ47829.1 |
| pY546-187 | - | 167,697-167,083 | 204 | hypothetical protein P821_05456 | EWD70887.1 |
| pY546-188 | - | 167,768-169,291 | 507 | group II intron reverse transcriptase/maturase | WP_064164938.1 |
| pY546-189 | - | 169,456-169,665 | 69 | hypothetical protein | WP_081112555.1 |
| pY546-190 | *tnp*A | 171,312-169,723 | 529 | TnpA | ADL13996.1 |
| pY546-191 | - | 171,692-171,342 | 116 | hypothetical protein ECs1338 | NP_309365.1 |
| pY546-192 | - | 172,096-171,689 | 135 | MULTISPECIES: hypothetical protein | WP_015632445.1 |
| pY546-193 | - | 172,172-172,336 | 54 | hypothetical protein SARI_03075 | YP_001572057.1 |
| pY546-194 | - | 173,702-172,569 | 377 | Zinc-containing alcohol dehydrogenase superfamily | YP_003741170.1 |
| pY546-195 | - | 175,726-174,161 | 521 | hypothetical protein P821_05453 | EWD70884.1 |
| pY546-196 | *IS*5/*IS*1182 | 175,983-176,906 | 307 | *IS*5/*IS*1182 family transposase | WP_032731993.1 |
| pY546-197 | - | 177,151-177,399 | 82 | hypothetical protein NT01EI_2008 | YP_002933419.1 |
| pY546-198 | *rel*E | 177,389-177,673 | 94 | MULTISPECIES: RelE toxin | WP_032731992.1 |
| pY546-199 | *ins*B | 178,228-178,566 | 112 | Insertion element *IS*1 protein InsB | EWD70880.1 |
| pY546-200 | - | 179,256-178,546 | 236 | hypothetical protein P821_05448 | EWD70879.1 |
| pY546-201 | *vap*C | 179,518-179,709 | 63 | tRNA(fMet)-specific endonuclease VapC | SFU18930.1 |
| pY546-202 | - | 179,986-180,396 | 136 | hypothetical protein P821_05447 | EWD70878.1 |
| pY546-203 | *uvr*A | 181,889-180,411 | 492 | excinuclease ABC subunit UvrA | WP_032732039.1 |
| pY546-204 | *uvr*A | 183,061-182,003 | 352 | MULTISPECIES: excinuclease ABC subunit UvrA | WP_023328883.1 |
| pY546-205 | - | 183,429-183,091 | 112 | hypothetical protein | WP_084227972.1 |
| pY546-206 | - | 183,586-183,467 | 39 | S-(Hydroxymethyl)glutathione dehydrogenase | EWD70876.1 |
| pY546-207 | - | 184,512-183,595 | 305 | S-(hydroxymethyl)glutathione dehydrogenase | EWD70875.1 |
| pY546-208 | - | 185,234-184,929 | 101 | MULTISPECIES: hypothetical protein | WP_023328886.1 |
| pY546-209 | - | 185,360-185,833 | 157 | MULTISPECIES: hypothetical protein | WP_032669225.1 |
| pY546-210 | - | 185,884-186,345 | 153 | MULTISPECIES: hypothetical protein | WP_032731991.1 |
| pY546-211 | *IS*5/*IS*1182 | 186,702-187,670 | 322 | *IS*5/*IS*1182 family transposase | WP_074440744.1 |
| pY546-212 | *IS*110 | 189,014-187,989 | 341 | MULTISPECIES: *IS*110 family transposase | WP_001101446.1 |
| pY546-213 | - | 190,909-189,758 | 383 | MULTISPECIES: hypothetical protein | WP_021567590.1 |
| pY546-214 | - | 191,891-190,929 | 320 | Zn-dependent protease | WP_050487753.1 |
| pY546-215 | - | 192,366-191,878 | 162 | hypothetical protein ECL_03698 | YP_003614181.1 |
| pY546-216 | - | 193,226-192,378 | 282 | hypothetical protein EL76_2415 | KGM61397.1 |
| pY546-217 | - | 193,399-193,292 | 35 | hypothetical protein EC54115_18267 | EIL49679.1 |
| pY546-218 | - | 195,102-193,396 | 568 | sodium/hydrogen exchanger | YP_002980594.1 |
| pY546-219 | - | 195,546-195,106 | 146 | MULTISPECIES: thiol reductase thioredoxin | WP_032731987.1 |
| pY546-220 | - | 196,141-195,536 | 201 | MULTISPECIES: hypothetical protein | WP_024146425.1 |
| pY546-221 | - | 196,769-196,098 | 223 | hypothetical protein A1UI_05061 | EOV71657.1 |
| pY546-222 | - | 196,974-196,783 | 63 | Uncharacterized protein | WP_004637111.1 |
| pY546-223 | - | 197,882-196,971 | 303 | conserved hypothetical protein | ZP_06726001.1 |
| pY546-224 | - | 198,640-197,966 | 224 | hypothetical protein Bmul_2303 | YP_001580485.1 |
| pY546-225 | - | 199,331-198,720 | 203 | MULTISPECIES: membrane protein | WP_032174377.1 |
| pY546-226 | - | 200,315-199,428 | 295 | MULTISPECIES: hypothetical protein | WP_021567627.1 |
| pY546-227 | - | 201,332-200,418 | 304 | MULTISPECIES: hypothetical protein | WP_021567628.1 |
| pY546-228 | *hsp*20 | 201,813-201,355 | 152 | MULTISPECIES: heat-shock protein Hsp20 | WP_032174845.1 |
| pY546-229 | *fts*H | 202,513-201,908 | 201 | ATP-dependent zinc metalloprotease FtsH | EQY56812.1 |
| pY546-230 | *fts*H | 203,051-202,458 | 197 | membrane protease FtsH catalytic subunit | ABE49436.1 |
| pY546-231 | - | 204,671-203,241 | 476 | cardiolipin synthase | WP_049129983.1 |
| pY546-232 | *cl*P | 207,549-204,688 | 953 | MULTISPECIES: ATP-dependent Clp protease ATP-binding subunit | WP_021567632.1 |
| pY546-233 | - | 208,219-207,647 | 190 | MULTISPECIES: Hsp20/alpha crystallin family protein | WP_032174387.1 |
| pY546-234 | - | 208,410-208,658 | 82 | hypothetical protein HMPREF9540_00418 | ZP_07133265.1 |

^*^ aa, amino acid.
